# Sleep Patterns, Genetic Susceptibility, and Incident Chronic Kidney Disease: A Prospective Study of 370 671 Participants

**DOI:** 10.3389/fnins.2022.725478

**Published:** 2022-01-31

**Authors:** Haojie Zhang, Bin Wang, Chi Chen, Ying Sun, Jie Chen, Xiao Tan, Fangzhen Xia, Jihui Zhang, Yingli Lu, Ningjian Wang

**Affiliations:** ^1^Institute and Department of Endocrinology and Metabolism, Shanghai Ninth People’s Hospital, Shanghai Jiao Tong University School of Medicine, Shanghai, China; ^2^Sleep Assessment Unit, Department of Psychiatry, Faculty of Medicine, The Chinese University of Hong Kong, Shatin, Hong Kong SAR, China; ^3^Department of Neuroscience, Uppsala University, Uppsala, Sweden; ^4^Department of Clinical Neuroscience, Karolinska Institutet, Stockholm, Sweden; ^5^Guangdong Mental Health Center, Guangdong Provincial People’s Hospital, Guangdong Academy of Medical Sciences, Guangzhou, China

**Keywords:** sleep pattern, gene, chronic kidney disease, prospective cohort, middle-aged and elderly population

## Abstract

**Objectives:**

Unhealthy sleep behaviors may be potential risk factors for chronic kidney disease (CKD). We aimed to examine the associations of combined sleep patterns and genetic susceptibility with incident CKD.

**Methods:**

This large-scale prospective cohort study included 370,671 participants without CKD at baseline (2006–2010) in UK Biobank data. Five sleep behaviors were made up of sleep duration, insomnia, snoring, chronotype, and daytime sleepiness according to questionnaire. Overall sleep patterns by summing the five scores were created. Weighted genetic risk score of kidney function was calculated. Incident CKD was recorded from death register, primary care, and hospital inpatient records. A subset of 41,130 individuals who participated both the initial assessment visit and follow-up visit (2012+) was also used.

**Results:**

During a median follow-up of 10.6 years (about 3.9 million person-years), we documented 6,365 patients with incident CKD. In five sleep behaviors, sleep 7–8 h/day, free of insomnia and no frequent daytime sleepiness were independently associated with incident CKD, with a 12% (95%CI 7–16), 9% (3–14), 13% (9–18) lower risk, respectively. Compared to those with a sleep score of 0–1, participants with a score of 5 had a 21% (10–31%) lower risk of CKD. 17.1% of CKD in this cohort could be attributed to total poor sleep pattern. Participants with high genetic risk and intermediate or poor sleep pattern showed the highest risk of CKD (OR = 2.58, 95%CI 2.24–2.96; OR = 2.59, 95%CI 2.02–3.32, respectively), although there was no significant interaction between sleep patterns and genetic risk categories. Among individuals who participated both the initial assessment visit and follow-up visit, we found that the association between amelioration of sleep pattern and risk of CKD was significant after fully adjustment (OR = 0.60, 95%CI 0.36–0.99), compared with group of stable sleep pattern.

**Conclusion:**

In this large prospective study, participants with a healthy sleep pattern was associated with a significant reduction of incident CKD risk no matter they had a high, intermediate, or low genetic risk.

## Introduction

Chronic kidney disease (CKD) has been becoming a critical public health burden worldwide, affecting about 10–15% of the world’s adult population and its prevalence is expected to rise further in the near future (Hill et al., 2016; Collaboration GBDCKD, 2020). All stages of CKD are linked to increased risks of cardiovascular disease, premature mortality, and decreased quality of life, thus, the identification of preventable causes of CKD are important medical problems (Hill et al., 2016).

Besides traditional lifestyle behaviors, emerging evidence has indicated some unhealthy sleep behaviors were important potential risk factors for CKD. For example, short sleep duration and insomnia was significantly associated with steeper decline in eGFR (Ricardo et al., 2017) and/or increased risk of CKD (Park et al., 2020). A link was also discovered between obstructive sleep apnea and stage 3 CKD or higher (Full et al., 2020). However, most of previous studies assessed the association of individual sleep behaviors with CKD and hardly consider the complexity and correlation of multiple sleep behaviors. In fact, as suggested in previous studies, sleep behaviors such as sleep duration, chronotype, insomnia, snoring and excessive daytime sleepiness are usually correlated and may impact human health and life quality in a joint manner (Fan et al., 2020). It seems only one cross-sectional study combined four sleep behaviors and indicated that worse overall sleep quality was not significantly associated with higher odds of CKD (Li et al., 2017). Therefore, due to partially controversial results, limited sleep behaviors, and no prospective cohort combining sleep behaviors, the available evidence is rather limited.

Genetic susceptibility and postnatal factors lead to the development of chronic diseases. Some studies have shown that lifestyles may interact with genetic predisposition on risk of CKD, which means even in population with high genetic susceptibility for CKD, healthy lifestyle such non-smoking may partly offset the later life risk for CKD (Hishida et al., 2014; Hannan et al., 2020). However, it is largely unclarified whether sleep behaviors, no matter single or combined, have interaction effects with genetic predisposition on the development of CKD.

In this prospective cohort of 370,671 participants from the UK Biobank, we measured the association between a healthy sleep score combing multiple sleep behaviors and incident CKD. We also estimated the proportion (population attributable risk, PAR%) of CKD events that theoretically would not have occurred if all participants had a healthy sleep pattern. We further examined the modification effect of genetic susceptibility combining 263 single nucleotide polymorphisms (SNPs) associated with CKD in this association.

## Materials and Methods

### Study Design and Sample

The UKB is a prospective cohort study that included more than 500,000 community-dwelling adults, aged 40--69 years, across the United Kingdom between 2006 and 2010^1^. A detailed information has been described in a previous study (Sudlow et al., 2015). We declare that all data are publicly available in the UKB repository (Sudlow et al., 2015). The North West Multi-Center Research Ethics Committee Study approved the UKB study, and all participants provided a written informed consent.

A total of 502,505 participants were recruited. We excluded those with missing values on any component of estimated glomerular filtration rate (eGFR) (age, sex, race, and creatinine) (*n* = 33,148), those with CKD at baseline (*n* = 20,660) and those with missing sleep variables (*n* = 78,026). The final sample was 370,671 in the main analyses. To further explore whether sleep pattern change would be associated with CKD risk, we used a subset of individuals who participated both the initial assessment visit (2006–2010) and follow-up visit (2012+). This sample size was 41,130.

### Exposure and Outcome

Self-reported information on sleep behaviors were collected at baseline recruitment. Detailed questions about the sleep behaviors have been described in Supplementary Files (Supplementary Table 1). Five sleep behavior components were used to define sleep pattern including sleep duration, chronotype, insomnia, snoring, and excessive daytime sleepiness. Based on previous studies (Wang et al., 2020) and the association between sleep categories and CKD (Supplementary File), “sleep behaviors with low risk were defined as follows: sleep 7–8 h/day; early chronotype (“morning” or “morning than evening”); reported never or rarely insomnia symptoms; no self-reported snoring; and no excessive daytime sleepiness (“never/rarely”).” We also coded sleep behaviors with low and high risk as 1 and 0 point, respectively. Then, we summed the five scores (0–5 points) to represent overall sleep pattern, with a higher score indicating a healthier one. We further divided the overall sleep patter into healthy (score 4–5), intermediate (score 2–3) and poor (score 0–1) sleep pattern. We also constructed a weighted sleep score according to the five sleep factors by using the equation: (ß1*factor1+…β5*factor5)/(5/β1+…+β5). This weighted score reflects magnitudes of the adjusted Odds Ratio for each sleep behavior in the combination of sleep patterns.

A subset of individuals provided sleep behavior information during the initial assessment visit (2006–2010) and follow-up visit (2012+). We defined stable sleep pattern as that total sleep score was the same as the baseline, and ameliorated or deteriorated sleep pattern as that total sleep score increased or decreased more than one point. A similar method was applied in each sleep component.

The outcome, CKD (field ID 132033), was extracted from “first occurrence of health outcomes defined by a 3-character International Statistical Classification of Diseases and Related Health Problems 10th Revision code” (category ID in UKB 1712). The sources of CKD were according to death register, primary care, and hospital inpatient records. Incident CKD was determined as total recorded CKD excluding participants with baseline eGFR < 60 ml/min per 1.73 m^2^ or with the date of first recorded occurrence being before or within 3 months after the date of attending assessment center.

### Genetic Risk Score for Estimated Glomerular Filtration Rate

Detailed information of genotyping in UK Biobank was reported previously (Bycroft et al., 2018). The weighted genetic risk score (GRS) was created for eGFR using SNPs associated with eGFR at the genome-wide association significance in a meta-analysis of genome-wide association studies that do not include UK Biobank participants (Locke et al., 2015). The information of the selected 263 SNPs is listed in Supplementary Table 2. Individual SNP was coded as 0, 1, and 2 according to the number of risk alleles. The regression coefficient for each SNP was taken from the reported meta-analysis and (Locke et al., 2015). GRS was formulated as the sum of the number of eGFR-decreasing alleles at each locus multiplied by the respective regression coefficient (Wang et al., 2018). We determined whether participants were at high (quintile 5), intermediate (quintile 2–4), or low (quintile 1) genetic risk for CKD.

### Covariates

The following potential confounders were included in the analysis: age, sex, ethnicity (White/others), education (university or college degree/others), the Townsend index reflecting socioeconomic status (continuous); smoking status (current, ever, never), drinking status (drinks, continuous), physical activity at goal or not (≥150 min/week of moderate intensity, or ≥75 min/week of vigorous intensity, or an equivalent combination); weight status (BMI ≥ 25 kg/m^2^ or not), diabetes (yes/no), systolic blood pressure (continuous), use of blood pressure-lowering medications (yes/no), use of diabetes medications (yes/no), autoimmune diseases (yes/no), diabetes well controlled (yes/no) and hypertension well controlled (yes/no). Participants who use diabetes medications and HbA1c < 6.5% were defined as diabetes well controlled, and those who use blood pressure-lowering medications and both systolic blood pressure < 140 mmHg and diastolic blood pressure < 90 mmHg were defined as hypertension well controlled. If the covariate information was missing, we imputed mean values for continuous variables or used a missing-indicator approach for categorical variables.

### Statistical Analyses

Data analyses were performed using IBM SPSS Statistics, Version 25 (IBM Corporation, Armonk, NY, United States) and SAS 9.2 (SAS Institute, Cary, NC, United States). A *P* value < 0.05 indicated a statistical significance (two-sided). Baseline characteristics of the study population are reported as means or percentages by sleep scores. Cumulative cases of CKD were calculated during follow-up visits. Follow-up time was determined from the baseline date (date of attending assessment center) to the diagnosis of CKD, death, or censoring date (August 31, 2019), whichever came first. In sleep-change data analyses, the corresponding “baseline date” was moved to the date of attending follow-up visit.

Logistic regression model was used to estimate the Odds Ratio (OR) and 95% confidence interval (CI). Model 1 was adjusted for age, sex, ethnicity (White/others), education (university or college degree/others) and the Townsend index. Model 2 was further adjusted for smoking status (current, ever, never), drinking status (drinks, continuous variable), and physical activity (MET minutes per week, continuous). Model 3 was adjusted for terms in model 2, overweight and obesity (BMI ≥ 25 kg/m^2^), diabetes (yes/no), systolic blood pressure, use of blood pressure-lowering medications (yes/no), and use of diabetes medications (yes/no).

We first measured the association of sleep behaviors with incident CKD. To estimate the population-level risk attributable to sleep behaviors, the hypothetical PAR% was calculated. It is an estimate of the proportion of incident CKD in the study population during follow-up that theoretically would be prevented if all people were in the low-risk sleep behavior groups, assuming a causal relationship (Wacholder et al., 1994).

We then classified participants according to the joint categories of sleep patterns (healthy sleep pattern 4–5 points, intermediate sleep pattern 2–3 points, and poor sleep pattern 0–1 point) and genetic risk for CKD (low, intermediate and high). Using participants with healthy sleep pattern and low genetic risk as reference, multivariate ORs of CKD were obtained in the left categories. The interaction analysis between sleep score and genetic susceptibility to CKD was performed by using the likelihood ratio test comparing models with and without a cross-product term.

We further performed subgroup analyses to exam the relationship between a gradual increase in healthy sleep score and CKD stratified by sex (men or women), age (≥60 or <60 years), BMI (≥25 or < 25 kg/m^2^), diabetes (yes or no), hypertension (yes or no), and dyslipidemia (yes or no), respectively.

In sleep-change data analyses, we measured multivariable-adjusted ORs for CKD in those with ameliorated or deteriorated sleep pattern and factor, compared with participants with stable sleep pattern and factor.

Finally, because CKD is usually asymptomatic until later stages, the incident time of CKD used in the current analysis may be later than the actual onset time, thus our main results were analyzed by logistic regression. Moreover, in the sensitivity analyses, we further adjusted autoimmune diseases, diabetes well controlled and hypertension well controlled, and provided results from Cox model and restricted subjects with incident CKD to ≥1 year from the baseline to perform the regression, respectively. We also analyzed this association using weighted healthy sleep score.

## Results

According to healthy sleep score, the baseline characteristics in participants are shown in Table 1. Among 370,671 participants, a healthy sleep score of 5, 4, 3, 2, and 0–1 was found in 6.2, 25.7, 35.7, 23.2, and 9.3% of total subjects, respectively. Compared to those the lowest healthy sleep score (0–1), participants with higher sleep score were more likely to be women and had significantly lower BMI, systolic blood pressure, and prevalence of diabetes.

**TABLE 1:** Baseline characteristics of 370,671 participants according to healthy sleep score.

Baseline characteristics	Healthy sleep score				
	0–1	2	3	4	5
N (%)	34,298	86,048	132,258	95,157	22,910
Age at baseline, years	56.9 ± 7.9	56.8 ± 7.9	56.4 ± 8.0	55.8 ± 8.2	54.3 ± 8.5
Men,%	53.3	48.8	44.1	41.2	44.9
Body mass index, kg/m^2^	29.2 ± 5.4	28.0 ± 4.9	27.2 ± 4.6	26.5 ± 4.3	26.0 ± 4.2
White,%	92.0	93.9	95.2	95.7	95.0
University or college degree,%	27.3	29.7	33.1	36.7	40.6
Townsend deprivation index	−1.75 (−3.41, 1.36)	−2.08 (−3.61, 0.64)	−2.28 (−3.72, 0.22)	−2.40 (−3.78, −0.09)	−2.41 (−3.78, −0.14)
Smoker, never%	45.8	50.1	54.5	59.2	63.8
Drinks, per week	6.0 (0.0, 13.0)	6.0 (0.0, 13.0)	6.0 (0.0, 12.0)	6.0 (0.0, 11.0)	5.0 (0.0, 10.0)
Physical activity, at goal%	44.8	48.8	52.6	56.0	59.5
Diabetes,%	8.9	6.1	4.4	3.5	3.1
Systolic blood pressure, mmHg	140.6 ± 18.7	140.4 ± 18.8	139.7 ± 19.0	138.7 ± 19.1	137.7 ± 19.1
Antihypertensive medication,%	27.1	22.4	19.1	16.0	12.4
**Sleep behaviors,%**					
Sleep 7–8 h/day	19.6	46.9	71.1	93.9	100.0
Early chronotype	21.8	42.9	63.1	85.9	100.0
Never/rarely insomnia	3.1	8.6	17.8	38.1	100.0
No self-reported snoring	16.5	41.7	65.4	86.2	100.0
No frequent daytime sleepiness	25.3	59.9	82.6	95.8	100.0

*Mean ± SD or median (interquartile range) for continuous variables and percentage for categorical variables.*

During the median follow-up time of 10.6 years (about 3.9 million person-years), we documented 6,365 patients with incident CKD. Supplementary Table 3 shows the association of each sleep behavior and incident CKD in different models. In demographic factors-adjusted model, short (<7 h) and long (>8 h) sleep duration, insomnia (sometimes and usually), snoring and excessive daytime sleepiness (sometimes and often/always) were each associated with an increased risk of incident of CKD. And after fully adjusted for smoking, drinking, physical activity, overweight and obesity, systolic blood pressure, diabetes, use of blood pressure-lowering medications and use of diabetes medications, these associated remained significant except short sleep duration, insomnia (sometimes) and snoring.

When the five sleep factors were categorized into binary factors of low risk vs. high risk (reference group), low-risk sleep behaviors including sleep 7–8 h/day (OR 0.88, 95% CI 0.84–0.93), never/rarely insomnia (OR 0.91, 0.86–0.97), and no frequent daytime sleepiness (OR 0.87, 0.82–0.91) were independently associated lower risk of incident CKD in the fully adjusted model (Table 2, model 3). Additionally, compared to those who cannot achieve the goal of five low-risk sleep behaviors, people who kept five healthy sleep behaviors had a 21% (10–31) lower risk to develop CKD in the final model (Table 2, model 3).

**TABLE 2:** Multivariable-adjusted Odds Ratio (95% CIs) for CKD by low-risk sleep factors.

	% of 370,671 participants	Model 1	Model 2	Model 3	Population attributable risk,%
Sleep 7–8 h/day	68.4	0.83 (0.79, 0.88)*	0.84 (0.80, 0.89)*	0.88 (0.84, 0.93)*	4.8 (2.9 to 6.8)
Early chronotype	62.7	0.97 (0.92, 1.02)	0.98 (0.93, 1.03)	1.00 (0.94, 1.05)	0.2 (−1.7 to 2.1)
Never/rarely insomnia	24.6	0.88 (0.82, 0.94)*	0.88 (0.83, 0.94)*	0.91 (0.86, 0.97)*	8.5 (3.7 to 13.1)
No self-reported snoring	62.8	0.90 (0.85, 0.95)*	0.90 (0.86, 0.95)*	0.99 (0.94, 1.05)	0.4 (−1.7 to 2.5)
No frequent daytime sleepiness	76.5	0.80 (0.76, 0.85)*	0.82 (0.78, 0.87)*	0.87 (0.82, 0.91)*	1.1 (0.4 to 1.7)
Five healthy behaviors	6.2	0.70 (0.62, 0.80)*	0.71 (0.63, 0.81)*	0.79 (0.69, 0.90)*	17.1 (7.7 to 26.3)

Model 1 was adjusted for age, sex, ethnicity (White/others), education (university or college degree/others) and the Townsend index (continuous). *P < 0.05. Model 2 was further adjusted for smoking status (current, ever, never), drinking status (drinks, continuous variable), physical activity (at goal or not). Model 3 was adjusted for terms in model 2 and overweight and obesity (BMI ≥ 25 kg/m^2^), systolic blood pressure, diabetes (yes/no), use of blood pressure-lowering medications (yes/no) and use of diabetes medications (yes/no). Five sleep behaviors were included simultaneously in the model.

The PAR% for sleep factors were calculated separately and combinedly (Table 2). Compared with the corresponding high-risk sleep behavior, the PAR% of participants with low-risk sleep behavior ranged from 1.1% (excessive daytime sleepiness) to 8.5% (insomnia). When participants had an overall healthy sleep score of five, the PAR reached 17.1%, suggesting that more than fifteen percent of incident CKD in this cohort will not have occurred if all participants were with all five healthy sleep behaviors.

**FIGURE 1:**
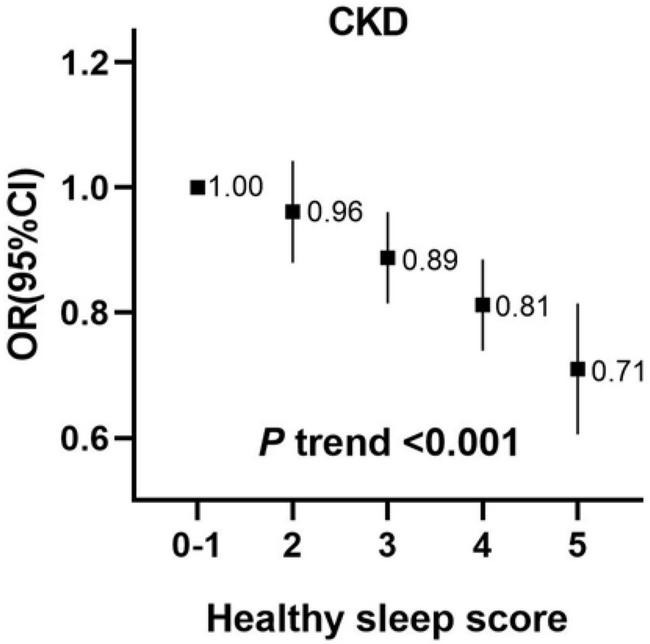
Incident risk of CKD according to healthy sleep score among 370,671 participants. The vertical line indicates the reference value of 1. Multivariable model was adjusted for age, sex, ethnicity (White/others), education (university or college degree/others) and the Townsend index (continuous), smoking status (current, ever, never), drinking status (drinks, continuous variable), physical activity (at goal or not), overweight and obesity (BMI ≥ 25 kg/m^2^), systolic blood pressure, diabetes (yes/no), use of blood pressure-lowering medications (yes/no) and use of diabetes medications (yes/no). CKD, chronic kidney disease; CI, confidence interval; OR, Odds Ratio.

When further combining the five binary sleep factors into a healthy sleep score, the risks of CKD decreased significantly with an increasing healthy sleep score (Figure 1, P for trend < 0.001). Compared to participants with a sleep score of 0–1, the fully adjusted ORs (95% CI) of those with a sleep score of 5 was 0.71 (0.61–0.82) for CKD. When measured ordinally, one increment of healthy sleep score was independently associated with an 8% lower risk of CKD (fully adjusted OR 0.92, 0.90–0.94).

**FIGURE 2:**
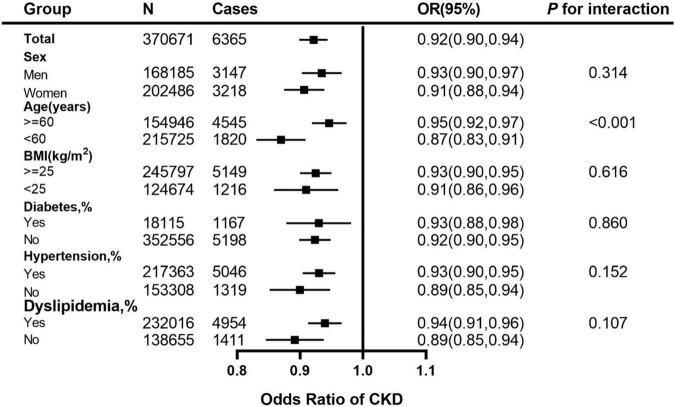
Adjusted ORs for healthy sleep score (per 1 score increment) associated with CKD in subgroups. The vertical line indicates the reference value of 1. The ORs with corresponding 95% CIs have been adjusted for age, sex, ethnicity (White/others), education (university or college degree/others) and the Townsend index (continuous), smoking status (current, ever, never), drinking status (drinks, continuous variable), physical activity (at goal or not), overweight and obesity (BMI ≥ 25 kg/m^2^), systolic blood pressure, diabetes (yes/no), use of blood pressure-lowering medications (yes/no) and use of diabetes medications (yes/no). CKD, chronic kidney disease; CI, confidence interval; OR, Odds Ratio.

We performed a stratified analysis, and further found that the associations for CKD with per 1 healthy sleep score increasing were broadly similar between both sexes and among other subgroups of levels of BMI and the status of diabetes, hypertension and dyslipidemia. Moreover, it seems that the association was modified by age categories (<60 years and ≥60 years) (Figure 2).

**FIGURE 3:**
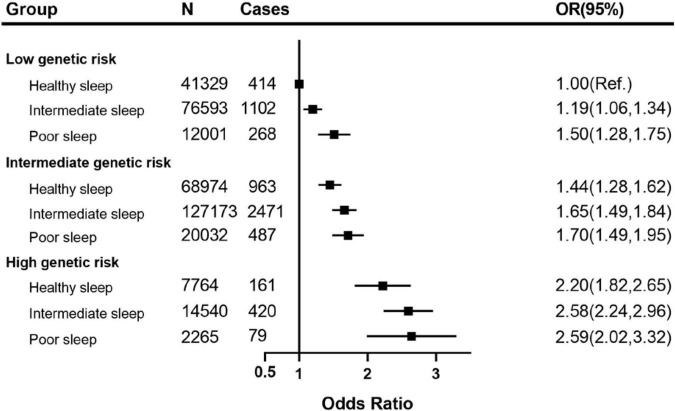
The joint association of weighted genetic risk and sleep pattern with CKD among 370,671 European ancestry participants. The vertical line indicates the reference value of 1. Multivariable model was adjusted for age, sex, ethnicity (White/others), education (university or college degree/others) and the Townsend index (continuous), smoking status (current, ever, never), drinking status (drinks, continuous variable), physical activity (at goal or not), overweight and obesity (BMI ≥ 25 kg/m2), systolic blood pressure, diabetes (yes/no), use of blood pressure-lowering medications (yes/no) and use of diabetes medications (yes/no). CKD, chronic kidney disease; CI, confidence interval; OR, Odds Ratio.

The joint associations between the healthy sleep score and polygenetic risk score of CKD are shown in Figure 3 (weighted GRS) and Supplementary Figure 2 (unweighted GRS). We found that compared with those with weighted low genetic risk and healthy sleep score, participants with high genetic risk and poor sleep pattern had a about 2.59-fold greater risk of CKD (OR 2.59, 2.02–3.32), even though there was no statistically significant interaction between healthy sleep score and genetic susceptibility to CKD (P for interaction = 0.115). A similar pattern of joint association was observed between healthy sleep score and unweighted genetic risk score for CKD (P for interaction = 0.560).

In sleep-change data analyses, we found that there was an inverse association between ameliorated sleep pattern and risk of CKD occurrence after fully adjustment (OR 0.60, 95%CI 0.36–0.99) and the association in deteriorated sleep pattern was positive, although non-significant (OR 1.18, 95% CI 0.80–1.76) (Supplementary Table 7). And in Supplementary Table 8, we observed that compared with stable sleep factor, participants with ameliorated sleep factor had lower risk of CKD and those with deteriorated chronotype, snoring and excessive daytime sleepiness had higher risk of CKD. This association was typically significant in worsening chronotype (OR 1.85, 95%CI 1.03–3.33). However, due to the small case sample size, we need view it with caution.

In sensitivity analyses, we have further adjusted autoimmune diseases, diabetes well controlled and hypertension well controlled, and the results remain stable (Supplementary Table 4). Then we further exerted Cox regression model to investigate the associations among CKD and five sleep patterns, and the results were largely unchanged (Supplementary Table 5). When limiting participants with a follow-up time over 1 year, the results also did not alter appreciably (Supplementary Table 6). The results were not materially changed for weighted healthy sleep score (Supplementary Figures 1, 3).

## Discussion

In this large-scale prospective cohort that included 370,671 middle-aged participants with 10.6-years follow-up time, the joint associations of a healthy sleep score combining five sleep behaviors (sleep duration, chronotype, insomnia, snoring, and excessive daytime sleepiness) and genetic predisposition with incident CKD were investigated. We found that compared with participants with sleep score of 0–1, those with a healthy sleep score of 5 had a 29% lower risk of developing CKD. Theoretically if causal, 17.1% of CKD could be prevented if all the participants had five good sleep behaviors. Moreover, participants with high genetic risk and poor sleep pattern had a about 2.6-fold greater risk of CKD compared with those with low genetic risk and healthy sleep pattern. The relationship between sleep patterns and CKD was further validated in sleep-change data analyses, and the significant association between improvement of sleep pattern and risk of CKD occurrence was found.

Sleep disorders are common in patients with CKD and associated with impairment of quality of life and increased mortality (Perl et al., 2006). It is reported that 50–80% of patients undergoing conventional hemodialysis experience certain symptoms of sleep disturbances, such as insomnia or excessive daytime sleepiness (Unruh et al., 2008). A prospective cohort study performed in a total of 3,600 participants with a 4.4 years median follow-up showed that insomnia during the night is associated with a moderately increased CKD risk, which was consistent with our main results (Sasaki et al., 2018). In concordance with our findings, several previous studies assessing individual sleep behavior indicated that short sleepers had a higher risk of proteinuria (Cheungpasitporn et al., 2017) and eGFR decline (Ricardo et al., 2017). Evidence also suggested that obstructive sleep apnea can contribute to the progression of CKD (Lin et al., 2020; Umbro et al., 2020). Additionally, Afsar (2013) found an independent association between excessive daytime sleepiness and 24-h urinary albumin excretion. On the contrary, other studies found that subjects with short sleep duration or poor sleep quality had a higher kidney infiltration rate relative to others (Chou et al., 2011; Petrov et al., 2014). These heterogenous findings are probably lie in differences of the study population, the relative sample size, and insufficient control of important confounders. Of note, most previous studies focused on the individual sleep behavior, and, to date, there is little data with regard to the association of combined sleep behaviors with CKD outcomes. It is of significance to investigate the combination of these sleep factors since these sleep-related behaviors are often interconnected. In the present study, we observed that a healthy sleep pattern was linked to a reduced risk of CKD events beyond conventional risk factors. Another aspect that deserves attention is the fact that we did not find a higher risk of incident CKD by late chronotype. However, in sleep-change data analyses, we found participants with worsening chronotype sleep component had a significant higher risk for incidence of CKD. Knowledge on the association between chronotype and CKD is still very limited and warrants further investigation.

What is the possible mechanism by which the combination of sleep behaviors might influence CKD risk? In fact, these sleep behaviors might individually act via several mechanisms that could operate synergistically to increase the risk of CKD. Experimental sleep deprivation has been revealed to result in increases in blood pressure and heart rate, enhanced salt retention, and alterations of glucose metabolism (Ricardo et al., 2017). Actually, in general population, short sleep duration predisposes individuals to hypertension (St-Onge et al., 2016), type 2 diabetes (Chaput et al., 2009; Shan et al., 2015) and coronary artery disease (Tobaldini et al., 2019), all of which are CKD risk factors. In addition, snoring, a symptom of sleep-related breathing disorder, has a notable adverse effect on the regulation of renin-angiotensin-aldosterone system (RAAS) via pathways of hypoxemia, oxidative stress, and activated sympathetic activities, which cause endothelial dysfunction and induce renal damage (Li et al., 2017; Lee et al., 2019).

As far as we know, this is the first prospective study to examine the joint association of sleep pattern and genetic risk score with incident CKD. We found that there was no statistically significant interaction effect between healthy sleep score and genetic susceptibility to CKD. Intriguingly, we observed that a high genetic predisposition could be partly offset by a healthy sleep pattern, but subjects with low genetic predisposition might lose their inherent protection if they had a poor sleep pattern. Accordingly, having a healthy sleep pattern is of critical importance in the primary prevention of CKD and its progression among the whole population regardless of genetic predisposition profile. Moreover, it is well known that the quality of sleep change profoundly across the lifespan (Carskadon et al., 1998), and sleep efficiency continued to significantly decrease after 60 years of age, especially (Ohayon et al., 2004). Our results found that participants under the age of 60 were more likely to have a reduced risk of CKD by keeping good sleep behaviors. Individuals under 60 have a healthier sleep pattern compared to their older counterparts, which may be associated with less activation of RAAS. RAAS activity not only leads to increased blood pressure, fluid retention, and positive sodium balance, but also to kidney damage by enhancing glomerular capillary filtration pressure and synthesis of profibrotic molecules such as transforming growth factor β (Koppe and Fouque, 2019). Hence, subjects under 60 may benefit more by sleep modification to prevent CKD occurrence or its progression.

The findings of ours and previous analyses suggest that modification of sleep behaviors might result in a better prognosis of renal function. In daily clinical practice, our findings underscore the importance of taking into account sleep behaviors in CKD management. From a public health perspective, the application of the simple score algorithm makes epidemiological findings easier to be understood and translated into practice, and hence to be informative to the general population.

The strengths of our study included a large sample size and relatively long follow-up duration. And there are few studies to exam the association between incident CKD and multiple sleep behaviors in large-scale cohort like us. Moreover, the novelty of our study is that we investigated the interaction between the genetic risk of CKD and sleep patterns on incident CKD, then we further performed subgroup analysis to reveal the stable association between sleep behaviors and CKD. Some limitations also remained in our study. First, this is an observational study, we cannot demonstrate the causal relationship between incident CKD and multiple sleep behaviors. Further randomized clinal trials are needed to reveal whether sleep modification could have a beneficial effect on renal function. Second, due to lack of follow-up blood data in incident CKD participants, CKD could not be graded specifically. Third, sleep behaviors were recorded by self-reported, and hence, classification error due to recall error could not be fully ruled out. However, as mentioned in previous studies (Li et al., 2015), the true association may be underestimated due to the presence of bias. Fourth, longtime follow-up sleep data should be needed because sleep pattern can change over time and be affected by several factors. However, we found a repetition rate of about 80% in more than 40,000 follow-up sleep data. Fifth, though we had adjusted for various major confounders carefully, bias from unknown and unmeasured confounding may still exist. Sixth, due to lack of the data of family history of CKD, drug overuses and history of acute kidney injury, we could not assess their impact on the result. Finally, this cohort included people of European descents, mostly White British, which limits the generalizability to other ethnicities, such as Asians and Blacks. UK Biobank aimed to be representative of the general population but was unrepresentative in terms of lifestyle because of a “healthy volunteer” selection bias (Fry et al., 2017). Therefore, generalizing summary statistics to the general population should be cautious.

In conclusion, in the present prospective population-based study of 370,671 individuals, participants with a healthy sleep pattern, including sleep 7–8 h per day, early chronotype, never or rarely insomnia, no snoring, and no frequent excessive daytime sleepiness, was associated with a significant reduction of incident CKD risk no matter they had a high, intermediate, or low genetic risk.

## Data Availability Statement

The raw data supporting the conclusions of this article will be made available by the authors, without undue reservation.

## Ethics Statement

The studies involving human participants were reviewed and approved by the North West Multi-Centre Research Ethics Committee Study approved the UKB study. The patients/participants provided their written informed consent to participate in this study.

## Author Contributions

NW was the guarantor of this work and, as such, had full access to all the data in the study and takes responsibility for the integrity of the data and the accuracy of the data analysis. HZ, CC, and NW wrote the manuscript, researched data, reviewed, and edited the manuscript. CC, BW, XT, JZ, and YL reviewed and edited the manuscript. JC, FX, and XT contributed to the discussion. All authors contributed to the article and approved the submitted version.

## Conflict of Interest

The authors declare that the research was conducted in the absence of any commercial or financial relationships that could be construed as a potential conflict of interest.

## Publisher’s Note

All claims expressed in this article are solely those of the authors and do not necessarily represent those of their affiliated organizations, or those of the publisher, the editors and the reviewers. Any product that may be evaluated in this article, or claim that may be made by its manufacturer, is not guaranteed or endorsed by the publisher.

## Footnotes

1
https://www.ukbiobank.ac.uk/
