# Supplementary material for: Sleep Patterns, Genetic Susceptibility, and Incident Chronic Kidney Disease: A Prospective Study of 370 671 Participants
Source: Front Neurosci. 2022 Jan 31;16:725478. doi: 10.3389/fnins.2022.725478 (PMC8843034; doi:10.3389/fnins.2022.725478)
Supplement: Supplementary file 1 [file Table_1.DOCX]

Sleep patterns, genetic susceptibility, and incident chronic kidney disease: a prospective study of 370,671 participants

Haojie Zhang, Bin Wang, Chi Chen, Ying Sun, Jie Chen, Xiao Tan, Fangzhen Xia, Jihui Zhang, Yingli Lu, Ningjian Wang

**Supplemental Table 1** Sleep questionnaire

**Supplemental Table 2** Components of the genetic risk score of CKD

**Supplemental Table 3** Multivariable-adjusted ORs (95%CIs) for incident CKD by sleep factors among 370671 participants

**Supplemental Table 4** Multivariable-adjusted Odds Ratio (95% CIs) for CKD by low-risk sleep factors, further adjusted for autoimmune diseases (yes/no), diabetes well controlled (yes/no) and hypertension well controlled (yes/no)

**Supplemental Table 5** Multivariable-adjusted HRs (95% CIs) for CKD by low-risk sleep factors using Cox regression model

**Supplemental Table 6** Multivariable-adjusted ORs (95% CIs) for CKD by low-risk sleep factors, limiting participants with a follow-up time of > 1 year

**Supplemental Table 7** Multivariable-adjusted Odds Ratio (95% CIs) for CKD by three total sleep patterns in 41,149 follow-up subjects.

**Supplemental Table 8** Multivariable-adjusted Odds Ratio (95% CIs) for CKD by each group of sleep component in 41,149 follow-up subjects.

**Supplemental Figure 1** Incident risk of CKD according to weighted healthy sleep score among 370671 participants

**Supplemental Figure 2** The joint association of unweighted genetic risk and sleep pattern with CKD among 370671 European ancestry participants

**Supplemental Figure 3** The joint association of weighted genetic risk and weighted sleep pattern with CKD among 370671 European ancestry participants

**Table 1 Sleep questionnaire**

| Sleep behavior | Questions | Field ID | Categories | Low risk behavior |
| --- | --- | --- | --- | --- |
| Sleep duration | About how many hours sleep do you get in every 24 hours? | 1160 | Normal (7–8 h), Short (<7 h), Long (>8 h) | Normal (7–8 h) |
| Chronotype | Do you consider yourself to be (1) definitely a “morning” person, (2) more a “morning” than “evening” person, (3) more an “evening” than “morning” person, or (4) definitely an “evening” person? | 1180 | Morningness, Morningness than eveningness, Eveningness than morningness, Eveningness | Morningness, Morningness than eveningness |
| Insomnia | Do you have trouble falling asleep at night or do you wake up in the middle of the night? If this varies a lot, answer this question in relation to the last 4 weeks. | 1200 | Never/rarely, Sometimes, Usually | Never/rarely |
| Snoring | Does your partner or a close relative or friend complain about your snoring? | 1210 | No, Yes | No |
| Daytime sleepiness | How likely are you to doze off or fall asleep during the daytime when you don’t mean to (e.g. when working, reading or driving)? | 1220 | Never/rarely, Sometimes, Often/Always | Never/rarely |

**Supplemental Table 2**. Components of the genetic risk score of CKD

| **SNP** | **EA** | **Chr** | **Effect** | **Locus** |
| --- | --- | --- | --- | --- |
| rs61830291 | a | 1 | -0.0036 | *LINC01352* |
| rs2490391 | a | 1 | -0.0024 | *SDCCAG8* |
| rs12061708 | a | 1 | -0.0026 | *KLHDC7A* |
| rs2749153 | a | 1 | -0.0033 | *ZNF436-AS1* |
| rs688540 | a | 1 | -0.0030 | *FOXD2* |
| rs3845534 | a | 1 | -0.0019 | *LOC100422212* |
| rs1011731 | a | 1 | -0.0019 | *DNM3* |
| rs78444298 | a | 1 | -0.0105 | *EDEM3* |
| rs78329830 | a | 1 | -0.0054 | *PLA2G4A* |
| rs1887252 | c | 1 | -0.0019 | *LINC01362* |
| rs7543734 | c | 1 | 0.0031 | *BCAR3* |
| rs74748843 | t | 1 | -0.0048 | *CASZ1* |
| rs659437 | t | 1 | -0.0027 | *AKR1A1* |
| rs10159261 | t | 1 | -0.0034 | *AGMAT* |
| rs267738 | t | 1 | -0.0048 | *CERS2* |
| rs17413465 | a | 1 | 0.0025 | *MIR4422HG* |
| rs11211257 | a | 1 | 0.0027 | *PIK3R3* |
| rs1757915 | a | 1 | 0.0021 | *LINC01755* |
| rs11166440 | a | 1 | 0.0020 | *CDC14A* |
| rs10857788 | a | 1 | 0.0030 | *SYPL2* |
| rs4971100 | a | 1 | 0.0020 | *TRIM46* |
| rs3850625 | a | 1 | 0.0046 | *CACNA1S* |
| rs2808454 | a | 1 | 0.0019 | *PFKFB2* |
| rs12736457 | c | 1 | 0.0054 | *PPM1J* |
| rs75625374 | c | 1 | 0.0045 | *CD34* |
| rs7536433 | t | 1 | 0.0021 | *AK5* |
| rs679843 | t | 1 | 0.0021 | *MGC27382* |
| rs3118119 | t | 1 | 0.0030 | *LOC105371433* |
| rs4656220 | t | 1 | 0.0020 | *PRRX1* |
| rs3795503 | t | 1 | 0.0020 | *KIAA1614* |
| rs7535253 | t | 1 | 0.0021 | *PTPN14* |
| rs2577134 | t | 1 | 0.0020 | *RNU5F-1* |
| rs417237 | t | 1 | 0.0018 | *OBSCN* |
| rs1047891 | a | 2 | -0.0065 | *CPS1* |
| rs17050272 | a | 2 | -0.0022 | *LINC01101* |
| rs2971880 | a | 2 | -0.0024 | *SPTBN1* |
| rs60980181 | a | 2 | -0.0027 | *CALCRL* |
| rs4664475 | t | 2 | -0.0020 | *NEB* |
| rs35472707 | t | 2 | -0.0073 | *LRP2* |
| rs2301343 | t | 2 | -0.0023 | *SLC8A1* |
| rs35284526 | a | 2 | 0.0029 | *NFE2L2* |
| rs4491726 | a | 2 | 0.0032 | *RDH14* |
| rs6546869 | a | 2 | 0.0059 | *ALMS1P1* |
| rs11694902 | a | 2 | 0.0041 | *TFCP2L1* |
| rs7425436 | a | 2 | 0.0024 | *ORC4* |
| rs35669853 | a | 2 | 0.0024 | *MIR5702* |
| rs10197255 | a | 2 | 0.0018 | *LINC01812* |
| rs10865189 | c | 2 | 0.0024 | *ZFP36L2* |
| rs187355703 | c | 2 | 0.0100 | *HOXD8* |
| rs780093 | t | 2 | 0.0044 | *GCKR* |
| rs11123169 | t | 2 | 0.0025 | *PSD4* |
| rs1548945 | t | 2 | 0.0036 | *TNP1* |
| rs1050816 | t | 2 | 0.0026 | *SPEG* |
| rs13003198 | t | 2 | 0.0018 | *SAG* |
| rs807624 | t | 2 | 0.0032 | *DDX1* |
| rs4666821 | t | 2 | 0.0020 | *PDE1A* |
| rs3791221 | a | 2 | 0.0022 | *SH3YL1* |
| rs6779998 | a | 3 | -0.0017 | *TGFBR2* |
| rs4625 | a | 3 | -0.0023 | *DAG1* |
| rs9828976 | c | 3 | -0.0024 | *SLC35G2* |
| rs56065557 | c | 3 | -0.0029 | *SENP2* |
| rs6778731 | t | 3 | -0.0017 | *WNT7A* |
| rs3774726 | t | 3 | -0.0021 | *ATXN7* |
| rs2289746 | t | 3 | -0.0019 | *CBLB* |
| rs35320690 | t | 3 | -0.0025 | *MSL2* |
| rs11919484 | t | 3 | -0.0026 | *KNG1* |
| rs2581820 | a | 3 | 0.0021 | *SFMBT1* |
| rs9868185 | a | 3 | 0.0026 | *SLC15A2* |
| rs1397764 | a | 3 | 0.0043 | *TFDP2* |
| rs9823161 | a | 3 | 0.0022 | *LINC02028* |
| rs11914389 | t | 3 | 0.0030 | *ACVR2B* |
| rs7651407 | t | 3 | 0.0025 | *PLXNB1* |
| rs10934754 | t | 3 | 0.0020 | *ALDH1L1-AS2* |
| rs7624084 | t | 3 | 0.0017 | *ZBTB38* |
| rs76272256 | t | 3 | 0.0024 | *MECOM* |
| rs795009 | t | 3 | 0.0020 | *SYN2* |
| rs3775932 | a | 4 | -0.0018 | *WDR1* |
| rs16874073 | t | 4 | -0.0045 | *PPARGC1A* |
| rs4864890 | t | 4 | -0.0023 | *DCUN1D4* |
| rs12509595 | t | 4 | -0.0035 | *FGF5* |
| rs75501914 | a | 4 | 0.0039 | *HGFAC* |
| rs71606723 | a | 4 | 0.0025 | *UGT8* |
| rs223471 | c | 4 | 0.0028 | *LOC102723704* |
| rs55929207 | c | 4 | 0.0019 | *ETNPPL* |
| rs28817415 | t | 4 | -0.0073 | *SHROOM3* |
| rs12163971 | a | 5 | -0.0029 | *AFF4* |
| rs13159523 | a | 5 | -0.0024 | *TPPP* |
| rs13157326 | a | 5 | -0.0027 | *RAI14* |
| rs2010352 | a | 5 | -0.0018 | *AK6* |
| rs1362800 | t | 5 | -0.0049 | *DAB2* |
| rs3812036 | t | 5 | -0.0065 | *SLC34A1* |
| rs72759880 | t | 5 | -0.0056 | *PIK3R1* |
| rs3797537 | a | 5 | 0.0019 | *DMGDH* |
| rs12520984 | c | 5 | 0.0019 | *FST* |
| rs12777 | c | 5 | 0.0050 | *SLC22A4* |
| rs11746506 | t | 5 | 0.0017 | *MRPS30* |
| rs11743174 | t | 5 | 0.0019 | *ABLIM3* |
| rs495237 | t | 5 | 0.0027 | *LINC00603* |
| rs79760705 | t | 5 | 0.0056 | *ARL15* |
| rs881858 | a | 6 | -0.0054 | *LINC01512* |
| rs72912510 | a | 6 | -0.0024 | *RRAGD* |
| rs9375818 | a | 6 | -0.0031 | *ARG1* |
| rs3822939 | a | 6 | -0.0025 | *EYA4* |
| rs12207180 | a | 6 | -0.0085 | *SLC22A2* |
| rs12212034 | t | 6 | -0.0018 | *PKHD1* |
| rs6458868 | t | 6 | -0.0020 | *GSTA2* |
| rs3925003 | t | 6 | -0.0018 | *HMGCLL1* |
| rs13200335 | a | 6 | 0.0024 | *TFEB* |
| rs11755724 | a | 6 | 0.0027 | *RREB1* |
| rs1857859 | a | 6 | 0.0019 | *SIM1* |
| rs1268168 | a | 6 | 0.0024 | *FOXO3* |
| rs9397738 | a | 6 | 0.0027 | *SCAF8* |
| rs77915916 | a | 6 | 0.0046 | *CRIP3* |
| rs7740107 | a | 6 | 0.0027 | *L3MBTL3* |
| rs3765502 | t | 6 | 0.0024 | *DCDC2* |
| rs144100226 | t | 6 | 0.0059 | *HMGA1* |
| rs720989 | t | 6 | 0.0021 | *SUPT3H* |
| rs35154268 | a | 7 | -0.0022 | *SND1* |
| rs6968554 | a | 7 | -0.0019 | *AHR* |
| rs62491533 | t | 7 | -0.0027 | *UBE2H* |
| rs10254101 | t | 7 | -0.0068 | *PRKAG2* |
| rs62435145 | t | 7 | -0.0060 | *UNCX* |
| rs3750081 | t | 7 | -0.0022 | *KBTBD2* |
| rs801193 | t | 7 | -0.0020 | *GS1-124K5.11* |
| rs6973656 | a | 7 | 0.0035 | *TMEM60* |
| rs700753 | c | 7 | 0.0031 | *LOC730338* |
| rs55773927 | t | 7 | 0.0019 | *VKORC1L1* |
| rs41301394 | t | 7 | 0.0023 | *POR* |
| rs3757387 | t | 7 | 0.0030 | *IRF5* |
| rs12671694 | t | 7 | 0.0025 | *SHH* |
| rs868822 | t | 7 | 0.0029 | *LINC01006* |
| rs11783418 | a | 8 | -0.0020 | *XKR6* |
| rs10102889 | c | 8 | -0.0036 | *NRG1* |
| rs2976178 | c | 8 | -0.0025 | *WWP1* |
| rs2980423 | t | 8 | -0.0023 | *PRAG1* |
| rs35353426 | t | 8 | -0.0026 | *LOC157273* |
| rs10098664 | t | 8 | -0.0021 | *BLK* |
| rs34861762 | t | 8 | -0.0043 | *STC1* |
| rs1533059 | a | 8 | 0.0025 | *MFHAS1* |
| rs7832708 | t | 8 | 0.0022 | *MSRA* |
| rs2954017 | t | 8 | 0.0024 | *TRIB1* |
| rs12377027 | a | 9 | -0.0026 | *MLLT3* |
| rs13287724 | a | 9 | -0.0030 | *B4GALT1-AS1* |
| rs1321917 | c | 9 | -0.0023 | *ASTN2* |
| rs544169 | a | 9 | 0.0022 | *UBAP2* |
| rs2039424 | a | 9 | 0.0044 | *PIP5K1B* |
| rs7024579 | t | 9 | 0.0023 | *QSOX2* |
| rs2068888 | a | 10 | -0.0024 | *CYP26A1* |
| rs4918943 | a | 10 | -0.0022 | *SORBS1* |
| rs12240572 | a | 10 | -0.0032 | *DNAJC9-AS1* |
| rs7095954 | a | 10 | -0.0018 | *TSPAN14* |
| rs816850 | c | 10 | -0.0020 | *KCNMA1* |
| rs1536225 | t | 10 | -0.0021 | *PDCD11* |
| rs7072591 | a | 10 | 0.0019 | *PARD3-AS1* |
| rs10821905 | a | 10 | 0.0037 | *A1CF* |
| rs1055256 | a | 10 | 0.0025 | *EEF1AKMT2* |
| rs80282103 | a | 10 | 0.0078 | *LARP4B* |
| rs6481598 | c | 10 | 0.0024 | *SVIL* |
| rs8474 | c | 10 | 0.0020 | *PARG* |
| rs7475348 | t | 10 | 0.0031 | *MYPN* |
| rs9420446 | t | 10 | 0.0023 | *FAM35A* |
| rs10821944 | t | 10 | 0.0020 | *ARID5B* |
| rs284859 | t | 10 | 0.0026 | *WBP1L* |
| rs1541937 | a | 11 | -0.0029 | *OR52H1* |
| rs1783827 | a | 11 | -0.0020 | *MIR130A* |
| rs3892895 | a | 11 | -0.0023 | *TPCN2* |
| rs963837 | t | 11 | -0.0057 | *DCDC1* |
| rs6484504 | t | 11 | -0.0026 | *DNAJC24* |
| rs2727040 | t | 11 | -0.0026 | *TRIM49B* |
| rs948493 | t | 11 | -0.0033 | *MIR1234* |
| rs11237450 | a | 11 | 0.0032 | *GAB2* |
| rs63934 | a | 11 | 0.0041 | *KCNQ1* |
| rs6589750 | a | 11 | 0.0020 | *USP2-AS1* |
| rs11564722 | t | 11 | 0.0033 | *INS-IGF2* |
| rs61897431 | t | 11 | 0.0029 | *SLC39A13* |
| rs7127946 | t | 11 | 0.0023 | *OR4B1* |
| rs1813937 | t | 11 | 0.0022 | *LOC646813* |
| rs10790452 | t | 11 | 0.0020 | *SORL1* |
| rs10846157 | a | 12 | -0.0034 | *RERG* |
| rs11062167 | a | 12 | -0.0039 | *SLC6A13* |
| rs632887 | a | 12 | 0.0032 | *TSPAN9* |
| rs117113238 | a | 12 | 0.0039 | *BCL2L14* |
| rs2634675 | a | 12 | 0.0025 | *ZNF641* |
| rs1275609 | a | 12 | 0.0024 | *PHLDA1* |
| rs4238020 | t | 12 | 0.0029 | *C12orf4* |
| rs12313306 | t | 12 | 0.0029 | *R3HDM2* |
| rs41284816 | t | 13 | -0.0078 | *DLEU2* |
| rs500830 | t | 13 | 0.0029 | *DACH1* |
| rs61993680 | a | 14 | -0.0019 | *SLC25A29* |
| rs72683923 | t | 14 | -0.0074 | *L2HGDH* |
| rs6574652 | t | 14 | -0.0017 | *STON2* |
| rs17184313 | t | 14 | -0.0029 | *RIN3* |
| rs1028455 | a | 14 | 0.0020 | *SPATA7* |
| rs690428 | a | 15 | -0.0039 | *WDR72* |
| rs1994887 | a | 15 | -0.0020 | *CGNL1* |
| rs351237 | a | 15 | -0.0018 | *STRA6* |
| rs4886696 | a | 15 | -0.0032 | *SIN3A* |
| rs6492982 | t | 15 | -0.0033 | *INO80* |
| rs11071738 | t | 15 | -0.0025 | *APH1B* |
| rs11071939 | t | 15 | -0.0039 | *SMAD3* |
| rs1145077 | t | 15 | -0.0085 | *GATM* |
| rs59646751 | t | 15 | -0.0023 | *IGF1R* |
| rs4886755 | a | 15 | 0.0041 | *NRG4* |
| rs17507300 | a | 15 | 0.0024 | *BTBD1* |
| rs7169629 | c | 15 | 0.0018 | *WDR73* |
| rs12913015 | t | 15 | 0.0027 | *C15orf54* |
| rs956006 | t | 15 | 0.0019 | *MGC15885* |
| rs2472297 | t | 15 | 0.0039 | *CYP1A1* |
| rs166906 | t | 15 | 0.0033 | *SCAPER* |
| rs9932625 | a | 16 | -0.0030 | *LINC01571* |
| rs28581385 | a | 16 | -0.0028 | *LINC01229* |
| rs154656 | a | 16 | -0.0030 | *CHMP1A* |
| rs1635404 | t | 16 | -0.0025 | *TRAP1* |
| rs193538 | t | 16 | -0.0020 | *ABCC1* |
| rs7185391 | t | 16 | -0.0027 | *SLC7A6* |
| rs62053077 | t | 16 | -0.0021 | *MARVELD3* |
| rs7203398 | a | 16 | 0.0025 | *CHD9* |
| rs77924615 | a | 16 | 0.0098 | *PDILT* |
| rs62050038 | a | 16 | 0.0028 | *WWP2* |
| rs438339 | t | 16 | 0.0035 | *RPL3L* |
| rs1858800 | t | 16 | 0.0020 | *ZFHX3* |
| rs883541 | a | 17 | -0.0022 | *PRKAR1A* |
| rs2411192 | a | 17 | -0.0024 | *MYO19* |
| rs8866 | c | 17 | -0.0018 | *PITPNC1* |
| rs2349648 | t | 17 | -0.0017 | *MPRIP* |
| rs4794813 | a | 17 | 0.0055 | *CDK12* |
| rs35662455 | c | 17 | 0.0030 | *TEX14* |
| rs9903801 | c | 17 | 0.0047 | *BCAS3* |
| rs9891340 | t | 17 | 0.0024 | *SMCR2* |
| rs2440165 | t | 17 | 0.0040 | *SLC47A1* |
| rs9895661 | t | 17 | 0.0069 | *BCAS3* |
| rs28735420 | t | 17 | 0.0039 | *MAP2K4* |
| rs227731 | t | 17 | 0.0018 | *C17orf67* |
| rs16942751 | a | 18 | -0.0029 | *AQP4* |
| rs1719934 | a | 18 | 0.0026 | *EPB41L3* |
| rs8096658 | c | 18 | 0.0050 | *NFATC1* |
| rs4940525 | t | 18 | 0.0025 | *LINC01544* |
| rs34647824 | a | 19 | -0.0021 | *RRAS* |
| rs78241494 | t | 19 | -0.0030 | *ZNF585A* |
| rs281380 | t | 19 | -0.0021 | *MAMSTR* |
| rs2974751 | a | 19 | 0.0018 | *CALR* |
| rs8101667 | t | 19 | 0.0044 | *CEP89* |
| rs7251730 | t | 19 | 0.0024 | *ZNF260* |
| rs113445505 | t | 19 | 0.0037 | *ZNF781* |
| rs6087579 | a | 20 | -0.0028 | *ITCH* |
| rs4408777 | a | 20 | -0.0021 | *RGS19* |
| rs2235826 | a | 20 | -0.0030 | *PCK1* |
| rs1041606 | t | 20 | -0.0021 | *MACROD2* |
| rs17216707 | t | 20 | -0.0051 | *CYP24A1* |
| rs1509117 | a | 20 | 0.0024 | *PLCB1* |
| rs72629024 | c | 20 | 0.0035 | *PPDPF* |
| rs62187537 | t | 20 | 0.0039 | FKBP1ASDCBP2 |
| rs1407040 | t | 20 | 0.0018 | *GNAS* |
| rs35636653 | t | 20 | 0.0022 | *OSBPL2* |
| rs2273684 | t | 20 | 0.0032 | *GSS* |
| rs2823139 | a | 21 | -0.0026 | *NRIP1* |
| rs2834317 | a | 21 | -0.0035 | *LOC101928126* |
| rs2244237 | t | 21 | 0.0027 | *CLDN14* |
| rs80576 | a | 22 | -0.0028 | *APOL3* |
| rs4820324 | c | 22 | -0.0023 | *MAFF* |
| rs131263 | t | 22 | 0.0024 | *ZMAT5* |
| rs112880707 | t | 22 | 0.0052 | *MKL1* |
| rs738527 | t | 22 | 0.0032 | *A4GALT* |

**Supplemental Table 3** Multivariable-adjusted ORs (95%CIs) for incident CKD by sleep factors among 370671 participants

|  | **N** | **Cases** | **Model 1** | **Model 2** | **Model 3** |
| --- | --- | --- | --- | --- | --- |
| **Sleep duration** |  |  |  |  |  |
| Normal (7–8 h) | 253396 | 3809 | 1.00 | 1.00 | 1.00 |
| Short (<7 h) | 89630 | 1653 | **1.08(1.12,1.15)** | **1.07(1.01,1.14)** | 1.06(0.99,1.12) |
| Long (>8 h) | 27645 | 751 | **1.39(1.28,1.51)** | **1.38(1.27,1.49)** | **1.27(1.17,1.38)** |
| **Chronotype** |  |  |  |  |  |
| Morningness | 100064 | 1858 | 1.00 | 1.00 | 1.00 |
| Morningness than eveningness | 132462 | 2161 | 0.98(0.92,1.05) | 0.98(0.92,1.04) | 1.00(0.94,1.06) |
| Eveningness than morningness | 105358 | 1670 | 1.00(0.94(1.07) | 0.99(0.93,1.06) | 0.99(0.93,1.06) |
| Eveningness | 32787 | 524 | 1.06(0.96,1.17) | 1.04(0.94,1.15) | 1.01(0.91,1.12) |
| **Insomnia** |  |  |  |  |  |
| Never/rarely | 91153 | 1223 | 1.00 | 1.00 | 1.00 |
| Sometimes | 177337 | 2878 | **1.10(1.02,1.17)** | **1.09(1.02,1.17)** | 1.06(0.99,1.14) |
| Usually | 102181 | 2112 | **1.28(1.18,1.38)** | **1.27(1.17,1.36)** | **1.18(1.09,1.27)** |
| **Snoring** |  |  |  |  |  |
| No | 232959 | 3695 | 1.00 | 1.00 | 1.00 |
| Yes | 137712 | 2518 | **1.10(1.05,1.16)** | **1.10(1.04,1.16)** | 1.00(0.95,1.05) |
| **Excessive daytime sleepiness** |  |  |  |  |  |
| Never/rarely | 283614 | 4149 | 1.00 | 1.00 | 1.00 |
| Sometimes | 77151 | 1767 | **1.21(1.14,1.28)** | **1.18(1.12,1.25)** | **1.13(1.07,1.20)** |
| Often/Always | 9906 | 297 | **1.48(1.31,1.68)** | **1.42(1.26,1.61)** | **1.28(1.13,1.45)** |

Model 1 was adjusted for age, sex, ethnicity (White/others), education (university or college degree/others) and the Townsend index (continuous).

Model 2 was further adjusted for smoking status (current, ever, never), drinking status (drinks, continuous variable), physical activity (at goal or not).

Model 3 was adjusted for terms in model 2 and overweight and obesity (BMI ≥25kg/m^2^), systolic blood pressure, diabetes (yes/no), use of blood pressure-lowering medications (yes/no) and use of diabetes medications (yes/no).

Five sleep behaviors were included simultaneously in the model.

**Supplemental Table 4** Multivariable-adjusted Odds Ratio (95% CIs) for CKD by low-risk sleep factors, further adjusted for autoimmune diseases (yes/no), diabetes well controlled (yes/no) and hypertension well controlled (yes/no)

|  | % of 370671 participants | Model 1 | Model 2 | Model 3 |
| --- | --- | --- | --- | --- |
| Sleep 7-8h/day | 68.4 | 0.83(0.79,0.88)* | 0.84(0.80,0.89)* | 0.88(0.84,0.93)* |
| Early chronotype | 62.7 | 0.97(0.92,1.02) | 0.98(0.93,1.03) | 1.00(0.95,1.05) |
| Never/rarely insomnia | 24.6 | 0.88(0.82,0.94)* | 0.88(0.83,0.94)* | 0.92(0.86,0.97)* |
| No self-reported snoring | 62.8 | 0.90(0.85,0.95)* | 0.90(0.86,0.95)* | 0.99(0.94,1.05) |
| No frequent daytime sleepiness | 76.5 | 0.80(0.76,0.85)* | 0.82(0.78,0.87)* | 0.87(0.82,0.92)* |
| Five healthy behaviors | 6.2 | 0.70(0.62,0.80)* | 0.71(0.63,0.81)* | 0.79(0.69,0.90)* |

Model 1 was adjusted for age, sex, ethnicity (White/others), education (university or college degree/others) and the Townsend index (continuous). * *P*<0.05

Model 2 was further adjusted for smoking status (current, ever, never), drinking status (drinks, continuous variable), physical activity (at goal or not).

Model 3 was adjusted for terms in model 2 and overweight and obesity (BMI ≥25kg/m^2^), systolic blood pressure, diabetes (yes/no), autoimmune diseases (yes/no), diabetes well controlled (yes/no) and hypertension well controlled (yes/no).

Five sleep behaviors were included simultaneously in the model.

**Supplemental Table 5** Multivariable-adjusted HRs (95% CIs) for CKD by low-risk sleep factors using Cox regression model

|  | % of 370671 participants | Model 1 | Model 2 | Model 3 |
| --- | --- | --- | --- | --- |
| Sleep 7-8h/day | 68.4 | 0.84(0.79,0.88)* | 0.84(0.80,0.89)* | 0.89(0.84,0.93)* |
| Early chronotype | 62.7 | 0.97(0.92,1.02) | 0.98(0.93,1.03) | 1.00(0.95,1.05) |
| Never/rarely insomnia | 24.6 | 0.88(0.83,0.94)* | 0.89(0.83,0.94)* | 0.92(0.86,0.98)* |
| No self-reported snoring | 62.8 | 0.90(0.86,0.95)* | 0.90(0.86,0.95)* | 0.99(0.94,1.05) |
| No frequent daytime sleepiness | 76.5 | 0.81(0.76,0.85)* | 0.82(0.78,0.87)* | 0.87(0.82,0.92)* |
| Five healthy behaviors | 6.2 | 0.70(0.62,0.80)* | 0.72(0.63,0.81)* | 0.79(0.69,0.90)* |

Model 1 was adjusted for age, sex, ethnicity (White/others), education (university or college degree/others) and the Townsend index (continuous). * *P*<0.05

Model 2 was further adjusted for smoking status (current, ever, never), drinking status (drinks, continuous variable), physical activity (at goal or not).

Model 3 was adjusted for terms in model 2 and overweight and obesity (BMI ≥25kg/m^2^), systolic blood pressure, diabetes (yes/no), use of blood pressure-lowering medications (yes/no) and use of diabetes medications (yes/no).

Five sleep behaviors were included simultaneously in the model.

**Supplemental Table 6** Multivariable-adjusted ORs (95% CIs) for CKD by low-risk sleep factors, limiting participants with a follow-up time of > 1 year.

|  | % of 370671 participants | Model 1 | Model 2 | Model 3 |
| --- | --- | --- | --- | --- |
| Sleep 7-8h/day | 68.4 | 0.83(0.78,0.87)* | 0.84(0.79,0.88)* | 0.88(0.83,0.93)* |
| Early chronotype | 62.7 | 0.95(0.90,1.01) | 0.97(0.91,1.02) | 0.99(0.93,1.04) |
| Never/rarely insomnia | 24.6 | 0.87(0.82,0.93)* | 0.88(0.82,0.94)* | 0.91(0.85,0.97)* |
| No self-reported snoring | 62.8 | 0.91(0.87,0.96)* | 0.92(0.87,0.97)* | 1.01(0.96,1.07) |
| No frequent daytime sleepiness | 76.5 | 0.80(0.75,0.84)* | 0.81(0.77,0.86)* | 0.86(0.81,0.91)* |
| Five healthy behaviors | 6.2 | 0.68(0.59,0.78)* | 0.69(0.60,0.80)* | 0.76(0.67,0.88)* |

Model 1 was adjusted for age, sex, ethnicity (White/others), education (university or college degree/others) and the Townsend index (continuous). * *P*<0.05

Model 2 was further adjusted for smoking status (current, ever, never), drinking status (drinks, continuous variable), physical activity (at goal or not).

Model 3 was adjusted for terms in model 2 and overweight and obesity (BMI ≥25kg/m^2^), systolic blood pressure, diabetes (yes/no), use of blood pressure-lowering medications (yes/no) and use of diabetes medications (yes/no).

Five sleep behaviors were included simultaneously in the model

**Supplemental Table 7** Multivariable-adjusted Odds Ratio (95% CIs) for CKD by three total sleep patterns in 41,149 follow-up subjects.

|  | Stable sleep pattern | Ameliorated sleep pattern | Deteriorated sleep pattern |
| --- | --- | --- | --- |
| Case/subject (%) | 54/17867 (0.3) | 22/10313 (0.2) | 47/12950 (0.4) |
| Odds Ratio | 1.00 (Ref.) | 0.60 (0.36,0.99) * | 1.18 (0.80,1.76) |

Multivariable model was adjusted for age, sex, ethnicity (White/others), education (university or college degree/others) and the Townsend index (continuous), smoking status (current, ever, never), drinking status (drinks, continuous variable), physical activity (at goal or not), overweight and obesity (BMI ≥25kg/m^2^), systolic blood pressure, diabetes (yes/no), use of blood pressure-lowering medications (yes/no) and use of diabetes medications (yes/no). * P = 0.045

**Supplemental Table 8** Multivariable-adjusted Odds Ratio (95% CIs) for CKD by each group of sleep component in 41,149 follow-up subjects.

|  | sleep duration | | chronotype | | insomnia | | snoring | | excessive daytime sleepiness | |
| --- | --- | --- | --- | --- | --- | --- | --- | --- | --- | --- |
|  | Incidence | Odds Ratio | Incidence | Odds Ratio | Incidence | Odds Ratio | Incidence | Odds Ratio | Incidence | Odds Ratio |
| Ameliorated sleep factor | 14/4735(0.3) | 0.88(0.50,1.55) | 6/2848(0.2) | 0.79(0.34,1.81) | 7/3346(0.2) | 0.59(0.27,1.27) | 10/4100(0.2) | 0.61(0.32,1.18) | 10/3475(0.3) | 0.80(0.42,1.55) |
| Stable sleep factor | 91/30306(0.3) | 1.00(Ref.) | 104/36265(0.3) | 1.00(Ref.) | 107/32368(0.3) | 1.00(Ref.) | 100/33159(0.3) | 1.00(Ref.) | 93/32989(0.3) | 1.00(Ref.) |
| Deteriorated sleep factor | 18/6089(0.3) | 0.92(0.55,1.53) | 13/2017(0.6) | 1.85(1.03,3.33)* | 9/5416(0.2) | 0.55(0.28,1.09) | 13/3871(0.3) | 1.30(0.72,2.33) | 20/4666(0.4) | 1.15(0.71,1.88) |

Case/subject (%) for incidence of CKD. Multivariable model was adjusted for age, sex, ethnicity (White/others), education (university or college degree/others) and the Townsend index (continuous), smoking status (current, ever, never), drinking status (drinks, continuous variable), physical activity (at goal or not), overweight and obesity (BMI ≥25kg/m^2^), systolic blood pressure, diabetes (yes/no), use of blood pressure-lowering medications (yes/no) and use of diabetes medications (yes/no). * P < 0.05


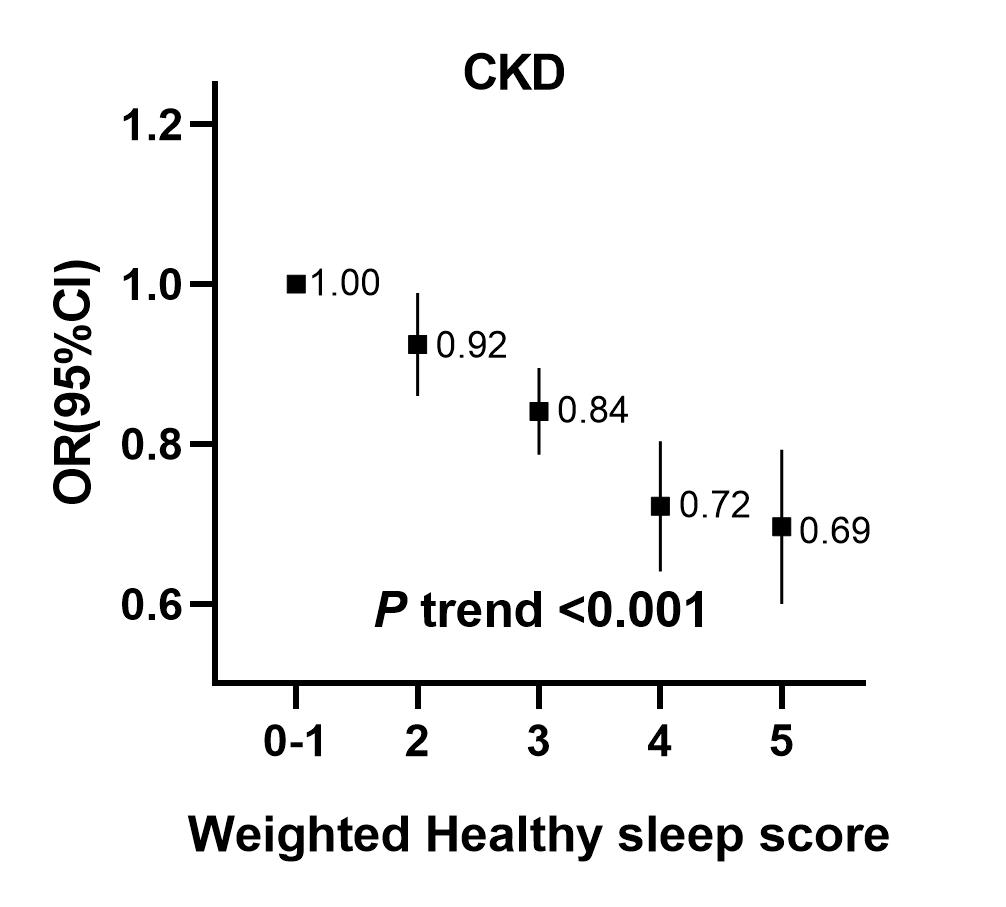


**Supplemental Figure 1** Incident risk of CKD according to weighted healthy sleep score among 370671 participants. The vertical line indicates the reference value of 1. Multivariable model was adjusted for age, sex, ethnicity (White/others), education (university or college degree/others) and the Townsend index (continuous), smoking status (current, ever, never), drinking status (drinks, continuous variable), physical activity (at goal or not), overweight and obesity (BMI ≥25kg/m^2^), systolic blood pressure, diabetes (yes/no), use of blood pressure-lowering medications (yes/no) and use of diabetes medications (yes/no). CKD, chronic kidney disease; CI, confidence interval; OR, Odds Ratio.


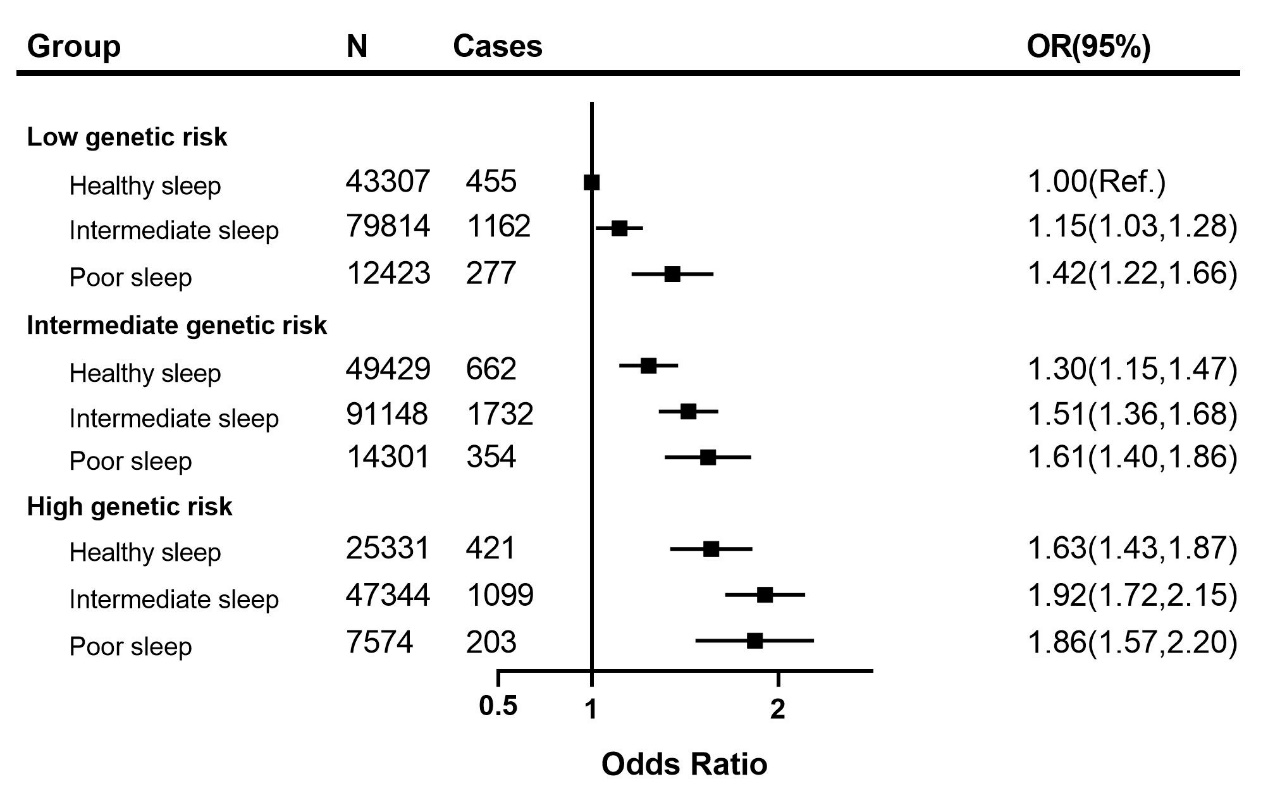


**Supplemental Figure 2** The joint association of unweighted genetic risk and sleep pattern with CKD among 370671 European ancestry participants. The vertical line indicates the reference value of 1. Multivariable model was adjusted for age, sex, ethnicity (White/others), education (university or college degree/others) and the Townsend index (continuous), smoking status (current, ever, never), drinking status (drinks, continuous variable), physical activity (at goal or not), overweight and obesity (BMI ≥25kg/m^2^), systolic blood pressure, diabetes (yes/no), use of blood pressure-lowering medications (yes/no) and use of diabetes medications (yes/no). CKD, chronic kidney disease; CI, confidence interval; OR, Odds Ratio.


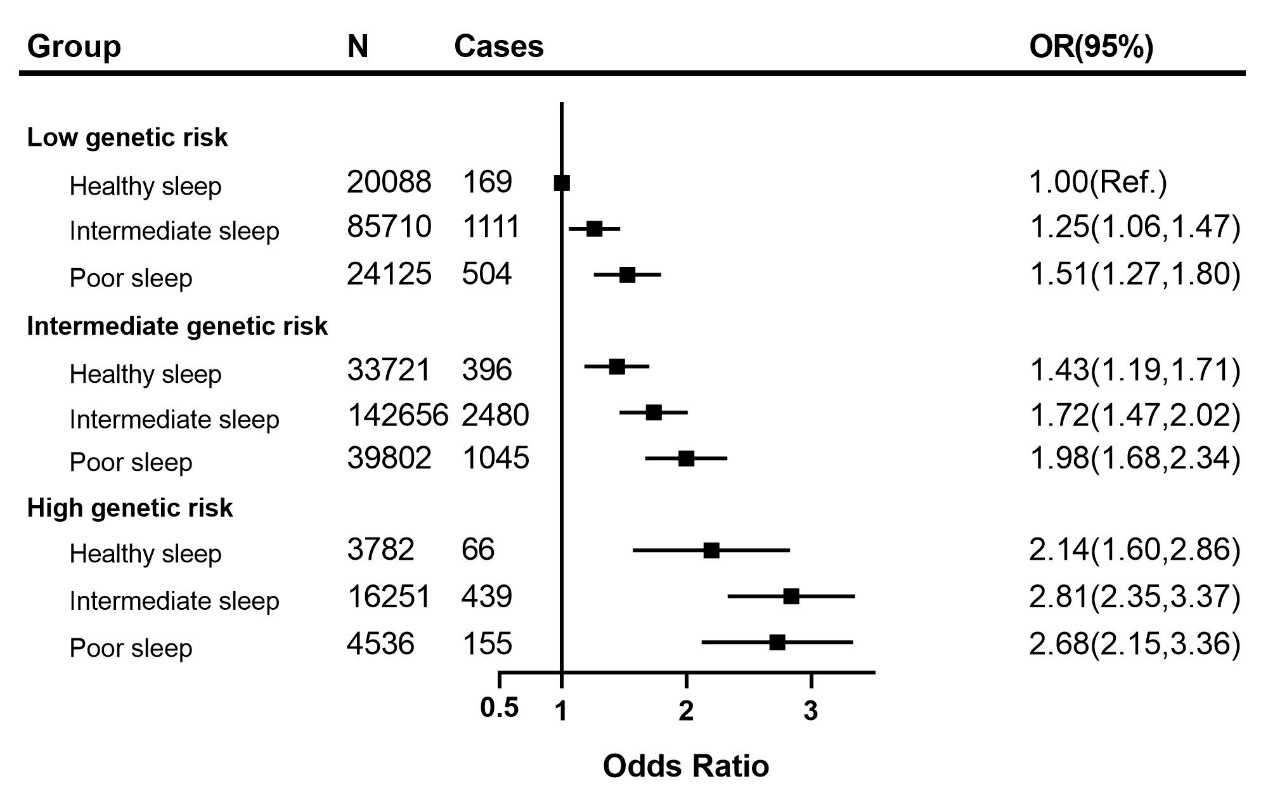


**Supplemental Figure 3** The joint association of weighted genetic risk and weighted sleep pattern with CKD among 370671 European ancestry participants. The vertical line indicates the reference value of 1. Multivariable model was adjusted for age, sex, ethnicity (White/others), education (university or college degree/others) and the Townsend index (continuous), smoking status (current, ever, never), drinking status (drinks, continuous variable), physical activity (at goal or not), overweight and obesity (BMI ≥25kg/m^2^), systolic blood pressure, diabetes (yes/no), use of blood pressure-lowering medications (yes/no) and use of diabetes medications (yes/no). CKD, chronic kidney disease; CI, confidence interval; OR, Odds Ratio.
